# Effects of Drugs Formerly Proposed for COVID-19 Treatment on Connexin43 Hemichannels

**DOI:** 10.3390/ijms23095018

**Published:** 2022-04-30

**Authors:** Axelle Cooreman, Anne Caufriez, Andrés Tabernilla, Raf Van Campenhout, Kaat Leroy, Prashant Kadam, Julen Sanz Serrano, Bruna dos Santos Rodrigues, Pieter Annaert, Mathieu Vinken

**Affiliations:** 1 Department of Pharmaceutical and Pharmacological Sciences, Vrije Universiteit Brussel, Laarbeeklaan 103, 1090 Brussels, Belgium; axelle.cooreman@vub.be (A.C.); anne.caufriez@vub.be (A.C.); andres.tabernilla.garcia@vub.be (A.T.); raf.van.campenhout@vub.be (R.V.C.); kaat.leroy@vub.be (K.L.); prashant.kadam@vub.be (P.K.); julen.sanz.serrano@vub.be (J.S.S.); bruna.dos.santos.rodrigues@vub.be (B.d.S.R.); 2 Drug Delivery and Disposition, Department of Pharmaceutical and Pharmacological Sciences, KU Leuven, 3000 Leuven, Belgium; pieter.annaert@kuleuven.be

**Keywords:** COVID-19, drug, connexin43, hemichannel, cellular communication

## Abstract

Connexin43 (Cx43) hemichannels form a pathway for cellular communication between the cell and its extracellular environment. Under pathological conditions, Cx43 hemichannels release adenosine triphosphate (ATP), which triggers inflammation. Over the past two years, azithromycin, chloroquine, dexamethasone, favipiravir, hydroxychloroquine, lopinavir, remdesivir, ribavirin, and ritonavir have been proposed as drugs for the treatment of the coronavirus disease 2019 (COVID-19), which is associated with prominent systemic inflammation. The current study aimed to investigate if Cx43 hemichannels, being key players in inflammation, could be affected by these drugs which were formerly designated as COVID-19 drugs. For this purpose, Cx43-transduced cells were exposed to these drugs. The effects on Cx43 hemichannel activity were assessed by measuring extracellular ATP release, while the effects at the transcriptional and translational levels were monitored by means of real-time quantitative reverse transcriptase polymerase chain reaction analysis and immunoblot analysis, respectively. Exposure to lopinavir and ritonavir combined (4:1 ratio), as well as to remdesivir, reduced Cx43 mRNA levels. None of the tested drugs affected Cx43 protein expression.

## 1. Introduction

Connexin hemichannels reside in the plasma membrane and are composed of six connexin (Cx) proteins. More than 20 connexin family members have been identified, all of which are expressed in a cell type-specific way and among which Cx43 is the most widespread variant. Connexin hemichannels have been considered for many decades as mere structural precursors of gap junctions, which mediate intercellular communication in a plethora of physiological processes [1]. However, in recent years, it has become clear that connexin hemichannels can provide a pathway for cellular communication on their own, albeit between the cytosol of an individual cell and its extracellular environment [2]. In contrast to gap junctions, connexin hemichannels have a low open probability. They seem to become preferentially activated by pathological stimuli, such as decreased extracellular calcium concentrations, mechanical stimulation, oxidative stress, ischemia/reperfusion injuries, and inflammatory conditions [3,4,5]. Among the messengers permeating connexin hemichannels, adenosine triphosphate (ATP) plays a key role in the induction and exacerbation of inflammation [6,7,8,9]. In this respect, inflammation is one of the hallmarks of the coronavirus disease 2019 (COVID-19). The first reported outbreak of the severe acute respiratory syndrome coronavirus 2 (SARS-CoV-2) took place in the Chinese province of Wuhan [10]. Since then, 470 million people worldwide have been infected with SARS-CoV-2, and as many as 6 million SARS-CoV-2-related deaths have been recorded as of March 2022 [11]. One of the most frequent complications of COVID-19 is pneumonia [12,13]. Severely ill patients can rapidly progress to acute respiratory distress syndrome within a week. This is observed in 3.6–17% of hospitalized patients with global mortality ranging from 3.4% to 88% for all COVID-19 cases [13,14,15,16,17,18]. Furthermore, several hospitalized COVID-19 patients develop sepsis, which is triggered by the host’s immune response to SARS-CoV-2 and leads to an aberrant inflammatory reaction or so-called cytokine storm [19]. In parallel with the worldwide efforts to generate prophylactic vaccines, an intensive search for drugs to treat COVID-19 was started early-on in the pandemic. Several existing drugs were proposed as COVID-19 therapeutics due to their anti-viral and/or anti-inflammatory effects, including azithromycin, chloroquine, dexamethasone, favipiravir, hydroxychloroquine, lopinavir, remdesivir, ribavirin, and ritonavir (Table 1) [12]. Given that connexin hemichannels, especially those built up by Cx43, play a role in inflammation, it seems conceivable to assume that connexin hemichannels are affected by drugs formerly regarded as COVID-19 drugs [1,20,21,22]. In fact, this defines the scope of the present study in which human embryonic kidney 293 (HEK) and Dubai camel (DUBCA) cells transduced with human Cx43 (hCx43) were exposed to azithromycin, chloroquine, dexamethasone, favipiravir, hydroxychloroquine, lopinavir, remdesivir, ribavirin, and ritonavir, as well as a combination of lopinavir and ritonavir, and their effects were investigated at the transcriptional, translational, and activity level of Cx43.

## 2. Results

### 2.1. Determination of Working Concentrations of the Drugs

**Figure 1:**
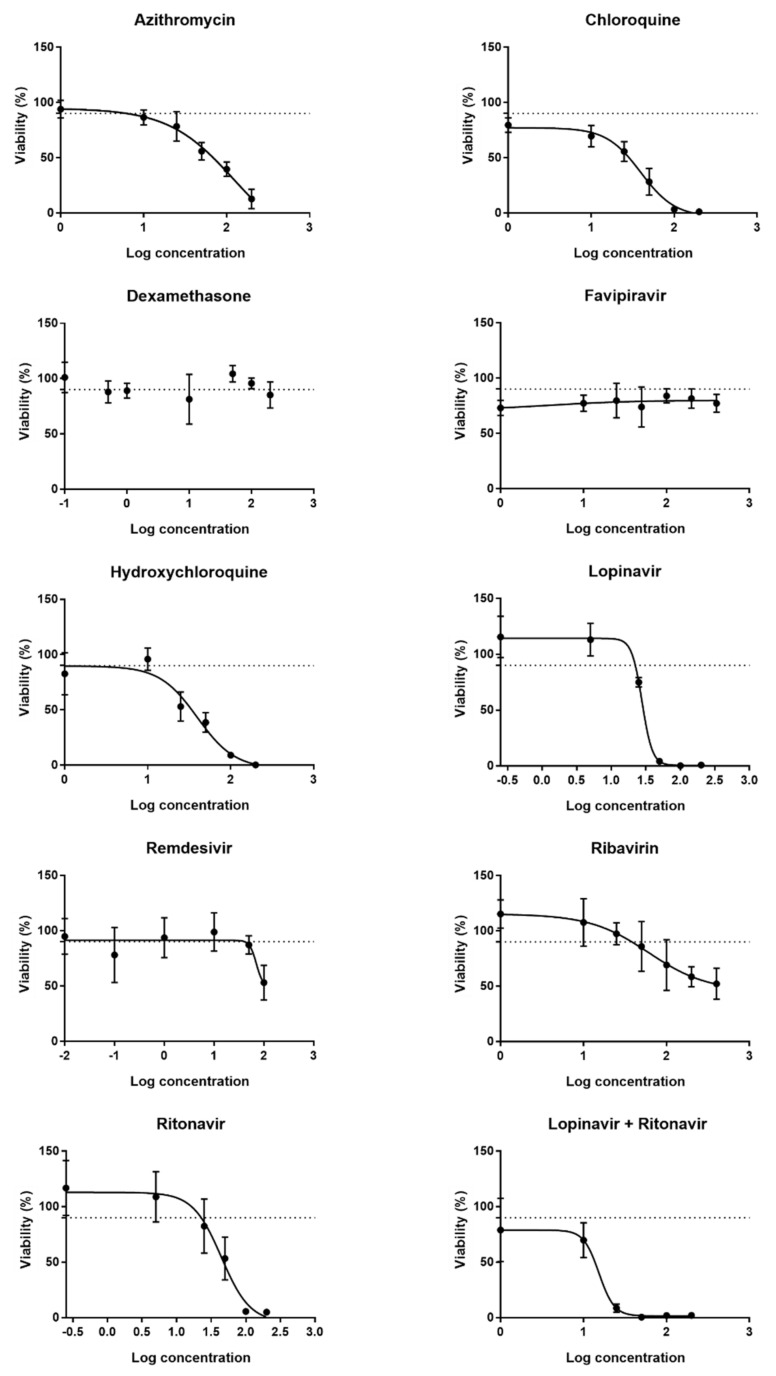
Concentration–response curves for the determination of the CC_10_ after 24 h of drug exposure of transduced Dubai camel cells overexpressing human Cx43 (DUBCA-hCx43). A sigmoidal curve was fitted by means of non-linear regression using GraphPad^®^ Prism to determine the CC_10_ value. Data are expressed as mean ± standard deviation (N = 4, *n* = 3).

In the first part of this study, a set of working concentrations of the drugs formerly proposed to treat COVID-19 was assessed by evaluating the effect of the drugs on cell viability using the 3-(4,5-dimethylthiazol-2-yl)-2,5-diphenyltetrazolium bromide (MTT) assay. DUBCA-hCx43 cells were exposed for 24 h to a broad range of concentrations of the drugs. For each drug, 6sixor seven different concentrations were selected, based on peak total plasma concentrations (C_max_) retrieved from the literature. Relevant in vitro concentrations are generally 10-fold higher than C_max_ values, therefore concentrations of this range were recalculated from the C_max_ values with a maximum of a 10-fold difference [32,33,34]. Data obtained by the MTT assay were used to generate concentration–response curves. Although the cytotoxic effects of the drugs on the cells should be preferably avoided, a certain fraction of the cells will spontaneously die following direct exposure to the compounds [35]. Hence, for each of the drugs, a benchmark concentration, inducing cell death in 10% of the cell population (CC_10_), was extrapolated from the concentration–response curves. Exposure of the cells to sequentially increasing concentrations of the drugs decreased cell viability gradually (data not shown). By non-linear regression of the obtained data set, sigmoidal curves were created (Figure 1), of which CC_10_ values could be extrapolated (Table 1). Dexamethasone and favipiravir did not show any cytotoxic effect on the cells. Consequently, concentration–response curves for these compounds could not be generated (Figure 1). In these cases, the C_max_ or a 10-fold of the C_max_ was used to arbitrarily set the CC_10_, ensuring a therapeutically relevant concentration range [25,26,36,37]. For the combination of lopinavir and ritonavir, the obtained CC_10_ value of lopinavir was used as a starting point since the addition of ritonavir solely contributes to the improvement of the pharmacokinetic properties of lopinavir [38]. A ratio of 4:1 was used to calculate the concentration for ritonavir, as this is proportional to the doses of both drugs administered to COVID-19 patients [39]. Next to the CC_10_, determined via the MTT assay or arbitrarily chosen for dexamethasone and favipiravir, the following concentrations were selected to be tested. This way, possible concentration-dependent effects of the drugs on Cx43 expression and/or activity could be evaluated. For testing the effects on Cx43 protein and mRNA levels, three different concentrations of the drugs were selected, namely CC_10_, CC_10_/2 and CC_10_/10. For assessing the effect of the drugs on Cx43 hemichannel activity, five different concentrations were selected, namely CC_10_ x10, CC_10_ x2, CC_10_, CC_10_/2 and CC_10_/10, since the exposure time of the cells to the drugs was shorter (30 min) compared to the exposure time to assess the CC_10_ via the MTT assay (24 h). Table 1 provides an overview of the drug concentrations that were applied in this study.

### 2.2. Effects of the Drugs on Cx43 Hemichannel Activity

**Figure 2:**
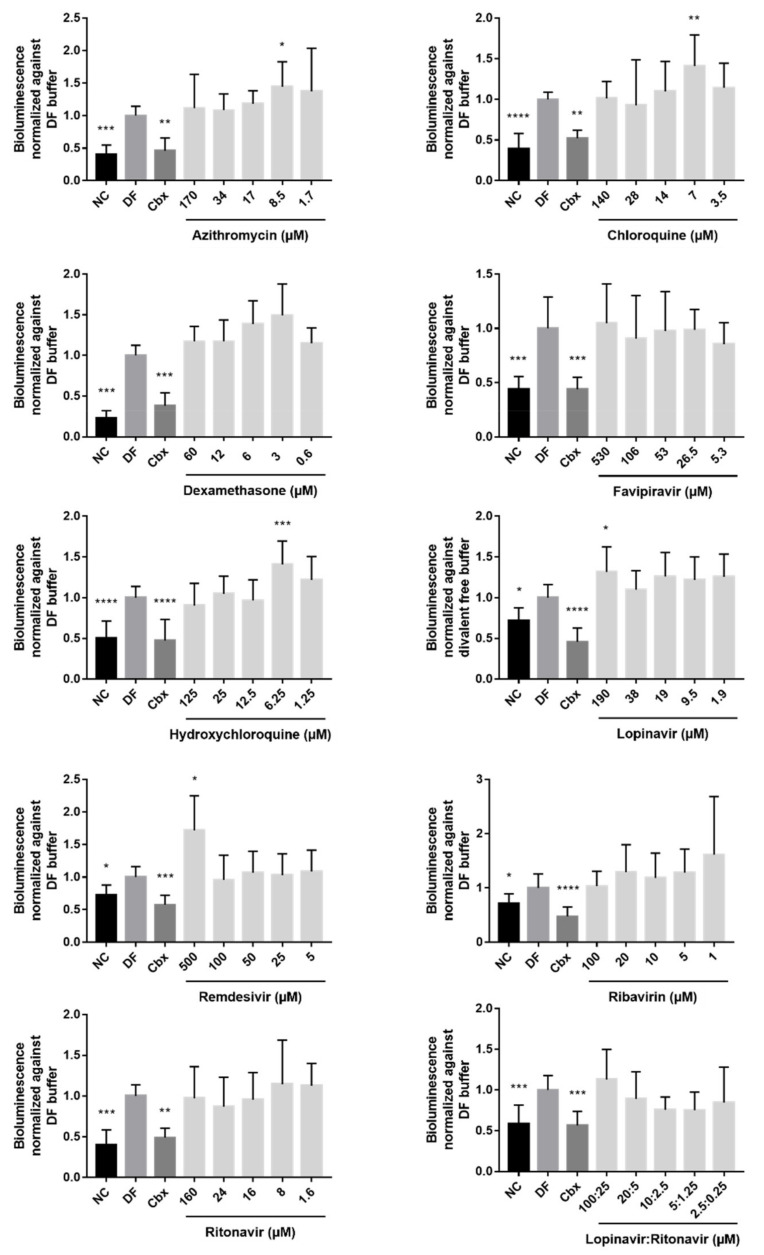
Effects of the drug panel on Cx43 hemichannel activity of transduced human embryonic kidney cells overexpressing human Cx43 (HEK-hCx43). The released amount of ATP of HEK-hCx43 cells exposed for 30 min to azithromycin, chloroquine, dexamethasone, favipiravir, hydroxychloroquine, lopinavir, remdesivir, ribavirin, ritonavir, and the combination of lopinavir and ritonavir, was measured. A buffer with normal calcium levels (NC) was used to mimic physiological conditions in which the hemichannels are typically closed. A buffer without divalent ions (DF) was used to open Cx43 hemichannels, while carbenoxolone (Cbx) dissolved in DF buffer was used as a Cx43 hemichannel inhibitor. Significant differences between the test conditions and the DF buffer were calculated with a parametric one-way analysis of variance or a non-parametric Kruskal–Wallis test followed by a Dunnett’s or Dunn’s multiple comparison test, respectively, depending on the distribution (i.e., Shapiro-Wilk normality test). Data are expressed as mean ± standard deviation with * *p* ≤ 0.05 ** *p* ≤ 0.01, *** *p* ≤ 0.001 and **** *p* ≤ 0.0001. (N = 4, *n* = 3) for azithromycin, chloroquine, dexamethasone, favipiravir, hydroxychloroquine, lopinavir, ribavirin and ritonavir. (N = 4; *n* = 4) for remdesivir. (N = 4; *n* = 5) for the combination of lopinavir and ritonavir.

Cx43 hemichannel activation is a frequent cellular response in pathological conditions accompanied by inflammation [40]. The RNA of the SARS-CoV-2 virus might act as a pathogen-associated molecular pattern (PAMP) [41]. Such PAMPs can trigger the opening of connexin hemichannels and thus release ATP in the extracellular environment [9]. Increased extracellular ATP levels activate purinergic P2X7 receptors at the cell membrane, which is a required step to further stimulate the pathway of inflammasome activation. Moreover, the opening of Cx43 hemichannels has been associated with the deterioration of the central neuronal processes in the progression of the pathogenesis of the human immunodeficiency virus (HIV) [42]. Altogether, this could suggest that drugs previously used to treat COVID-19 with anti-inflammatory or anti-viral effects could alter the opening of Cx43 hemichannels. Therefore, HEK-hCx43 cells were exposed to five different concentrations of each drug for 30 min (Table 1). Since the amount of ATP released in the extracellular environment is indirectly correlated with the activity of Cx43 hemichannels, ATP signals were measured via bioluminescence. Parallel with the exposure of the cells to the different drugs, cells were also exposed to three different controls. The first control condition included cells exposed to a buffer with physiological or normal calcium (NC) levels. The state of the Cx43 hemichannels of the cells exposed to the NC buffer is similar to their state in physiological conditions, i.e., in a closed or flickering state [43,44]. The second control condition implied exposure of the cells to a divalent free (DF) buffer. Creating an environment with decreased calcium levels forces Cx43 hemichannels to open and release high amounts of ATP in the extracellular milieu. The third control included exposure of the cells to carbenoxolone (Cbx), which is a general inhibitor of connexin-based channels [45]. Indeed, cells exposed to a NC buffer or Cbx showed lower ATP levels and hence lower Cx43 hemichannel activity compared to the cells exposed to a DF buffer (Figure 2). Extracellular ATP levels released by the HEK-hCx43 cells exposed to dexamethasone, favipiravir, ribavirin, ritonavir, and the combination of lopinavir and ritonavir were not significantly different from the DF control (Figure 2). However, the opposite seemed to occur when HEK-hCx43 cells were exposed to 8.5 µM azithromycin, 7 µM chloroquine, and 6.25 µM hydroxychloroquine, which are all CC_10_/2 concentrations of these drugs. In addition, starting from the CC_10_ x10 concentration and moving towards the CC_10_/2 concentration, the ATP levels were rising. A similar stepwise increase in extracellular ATP levels, upon the use of lower concentrations of the drugs, seemed to occur after exposure of the cells to dexamethasone. This suggests that dexamethasone might have a concentration-dependent effect on ATP release. Cells exposed to the highest applied concentrations of lopinavir and remdesivir, 190 and 500 µM, respectively, also released a significantly higher amount of ATP (Figure 2). Following the evaluation of the ATP release, the viability of the cells exposed to the drugs or controls was monitored with the MTT assay. None of the conditions showed a decreased cell viability compared to cells incubated with a DF buffer, indicating that cell viability was not interfering with the ATP levels and that the effects that were seen could be attributed to the compounds and not to any cytotoxic effect.

### 2.3. Effects of the Drugs on Cx43 mRNA and Protein Expression

**Figure 3:**
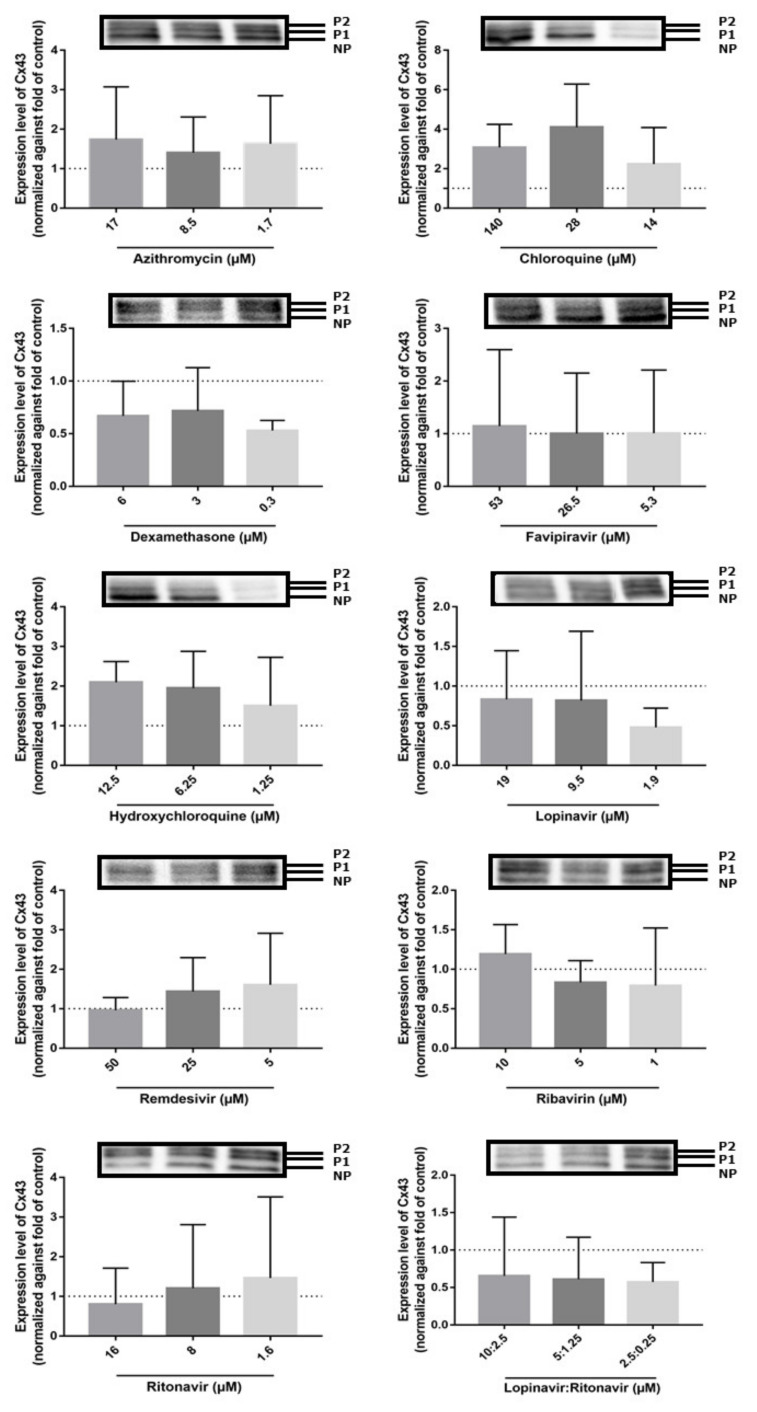
Cx43 protein expression after 24 h of drug exposure of transduced Dubai camel cells overexpressing human Cx43. Both the phosphorylated (P1 and P2) and non-phosphorylated (NP) variants could be detected. Signals of Cx43 were normalized against total protein loading and expressed as relative alterations compared to untreated controls (dashed line), using Image Lab software. Statistical analysis was performed using a parametric one-way analysis of variance or non-parametric Kruskal–Wallis test in combination with a Dunnett’s or Dunn’s test, depending on the normality of the data. Data are expressed as mean ± standard deviation (N = 1, *n* = 3).

**Figure 4:**
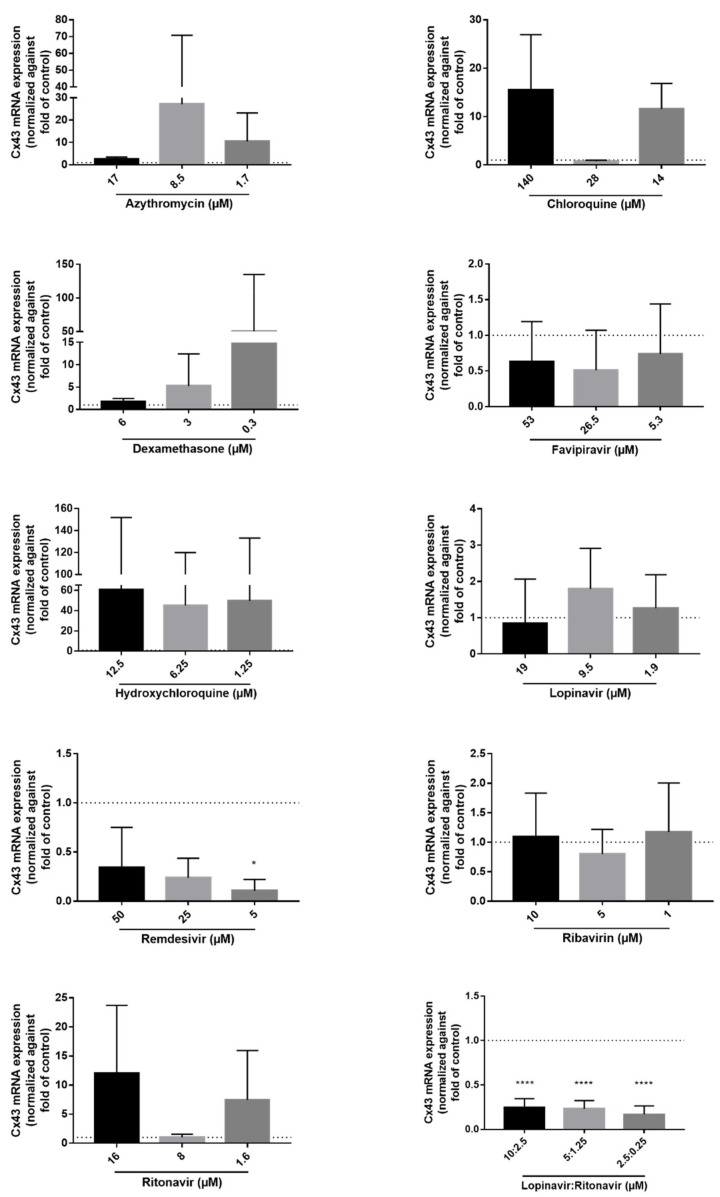
Cx43 mRNA expression after 24 h of exposure of transduced Dubai camel cells overexpressing human Cx43 to the drugs. mRNA expression levels were measured using RT-qPCR. Data were analyzed with the Pfaffl method and normalized against untreated controls (dashed line) [50]. Statistical analysis was performed using a parametric one-way analysis of variance or non-parametric Kruskal-Wallis test in combination with a Dunnett’s or Dunn’s test, respectively, depending on the normality of the data (i.e., Shapiro–Wilk normality test). Data are expressed as mean ± standard deviation with * *p* ≤ 0.05 and **** *p* ≤ 0.0001 (N = 3, *n* = 3).

Both Cx43 mRNA and protein expression levels are frequently upregulated in inflammatory conditions [40,46,47,48]. To assess if the former COVID-19 drugs alter Cx43 expression, transduced DUBCA-hCx43 cells were exposed to CC_10_, CC_10_/2 and CC_10_/10 concentration of the drugs for 24 h (Table 1). While semi-quantitative Cx43 protein expression was assessed by means of immunoblot analysis, RT-qPCR was used to evaluate Cx43 mRNA expression levels. Upon immunoblot analysis of the samples, three bands appeared for Cx43 at diverse molecular weights around 43 kD, representing the different existing phosphorylated isoforms. The lowest or fast-migrating band corresponds to the non-phosphorylated form of Cx43 (NP-Cx43), while two additional, slow-migrating bands appear at higher molecular weights corresponding to the phosphorylated isoforms of Cx43 (P1-Cx43 and P2-Cx43) (Figure 3) [49]. None of the drugs changed the Cx43 protein expression levels (Figure 3). DUBCA-hCx43 cells exposed to the combination of lopinavir and ritonavir tended to decrease Cx43 protein expression. Unlike the absence of statistically significant effects on the translational level, remdesivir and the combination of lopinavir and ritonavir triggered a decrease in Cx43 mRNA expression (Figure 4). For the combination of lopinavir and ritonavir, all the tested concentrations lowered Cx43 mRNA expression, while for remdesivir this effect was only seen at 5 µM. In line with the observations at the protein level (Figure 3), no effects of the other drugs (azithromycin, chloroquine, dexamethasone, favipiravir, hydroxychloroquine, lopinavir, ribavirin, and ritonavir) in any concentration on Cx43 mRNA quantities were noticed (Figure 4).

## 3. Discussion

COVID-19 is a viral infectious disease that can severely damage multiple organs and ultimately lead to death [12]. At different levels in the pathogenesis of COVID-19, Cx43 hemichannels can be involved in the initiation and dissemination of inflammatory processes. Recently, it was reported that Cx43 expression levels in human epithelial cells are upregulated upon exposure to SARS-CoV-2 [51]. SARS-CoV-2 starts causing damage to the cells at the pulmonary level, where the virus infects alveolar type II cells and alveolar macrophages, resulting in pneumonia and acute respiratory distress syndrome [12,52,53]. In acute respiratory distress syndrome, the influx of neutrophils can be related to the opening of Cx43 hemichannels. On the one hand, the release of ATP by Cx43 hemichannels in the extracellular milieu leads to direct stimulation of purinergic receptors at the cell plasma membrane, which in turn activates inflammasomes. On the other hand, the release of ATP in the extracellular milieu by Cx43 hemichannels at the surface of polymorphonuclear leukocytes promotes the endothelial barrier function but attenuates the adhesion between neutrophils and the endothelium [54,55]. Not only in the pulmonary phase, but equally in the subsequent phase, namely the pro-inflammatory stage of COVID-19, neutrophil activation is an important key event. In this stage, RNA virus-specific motifs, such as 5′-triphosphate and double-stranded RNAs of SARS-CoV-2, are recognized by the cells as PAMPs, which leads to the activation of the inflammatory pathway and the formation of active inflammasomes [12]. In COVID-19, this reaction results in an overproduction of cytokines, a recognized phenomenon in acute lung injury [12]. Using an in vivo model for acute lung injury, namely lipopolysaccharide-instilled mice, it was shown that Cx43 hemichannels contribute to neutrophil transmigration towards the airspace [56]. Collectively, this could suggest a role for Cx43 hemichannels in neutrophil activation and transmigration in COVID-19 pathogenesis [3,7,21,22,57]. Furthermore, platelet aggregation and thrombosis, which occurs in the prothrombic phase and final phase of COVID-19, can develop towards multi-organ failure with death as outcome [12]. On these three different levels in the pathogenesis of COVID-19, drugs are used to modulate and prevent further progression of COVID-19. The hypothesis of the current study was to investigate the possible effects of a number of these drugs, previously proposed to treat COVID-19, on Cx43 hemichannels. Based on the analysis of Cx43 hemichannel activity via assessment of extracellular ATP release, none of the drugs showed a significant inhibitory effect. In contrast, lower concentrations of azithromycin, chloroquine and hydroxychloroquine increase extracellular ATP levels (Figure 2). The combination of azithromycin and hydroxychloroquine and hydroxychloroquine alone induced an elevation of Cx43 protein expression in cardiomyocytes as a result of increased toxicity because of the intracellular accumulation of these drugs [58]. Chloroquine has similar effects on Cx43 expression, being an inhibitor of autophagic degradation [59]. Nevertheless, azithromycin, chloroquine, and hydroxychloroquine did not evoke any changes at the Cx43 expression level. Moreover, these findings are not in line with the immunomodulatory capacities of chloroquine and hydroxychloroquine to reduce the production of pro-inflammatory cytokines such as TNF-α and IL-6 [60]. Cells exposed to remdesivir and the combination of lopinavir and ritonavir displayed decreased levels of Cx43 mRNA (Figure 4). The combination of lopinavir and ritonavir is an approved treatment for acquired immunodeficiency syndrome. In this regard, it has been reported that Cx43 hemichannels are involved in the pathogenesis of HIV and altered effects on Cx43 hemichannels were seen both at the activity and expression levels [42,61]. This supports the hypothesis that lopinavir and ritonavir decrease Cx43 mRNA expression and counteract inflammation (Figure 4). In contrast, others found elevated Cx43 expression levels in HEK cells treated with lopinavir and ritonavir [62]. Remdesivir also has a decreasing effect on Cx43 mRNA expression. Lopinavir, ritonavir, and remdesivir all interfere with the progression of COVID-19 already in the pulmonary phase [12]. However, the mechanism of action is slightly different for these drugs. While remdesivir inhibits viral RNA transcription, lopinavir and ritonavir tackle the inhibition of the viral proteases [63,64]. Alterations in the mRNA level upon exposure to these drugs were not confirmed at the activity level nor at the protein level. Dexamethasone, favipiravir and ribavirin did not show any effect on Cx43 and its hemichannels at any level. Next to the effect of these drugs on hemichannel activity, it can be of relevance to investigate the effects of these drugs on gap junctional communication, since this can be reduced in pathological conditions [4]. Indeed, dexamethasone was found to reduce gap junctional communication [65,66]. Chloroquine, on the other hand, did not had any effects on gap junctional communication [67,68]. Since the start of this study, new drugs and/or their combinations were authorized by the European Medicines Agency to treat COVID-19, since the formerly proposed drugs did not show the desired effects or were causing adverse effects [69,70,71,72,73,74,75]. In the new list of seven compounds authorized for COVID-19 treatment in European Union (anakinra, PF-07321332/ritonavir, remdesivir, regdanvimab, tocilizumab, casirivimab/imdevimab, and sotovimab), four compounds (regdanvimab, tocilizumab, casirivimab/imdevimab, and sotovimab) are monoclonal antibodies directed against the receptor-binding domain of the SARS-CoV-2 glycoprotein. In this way, the SARS-CoV-2 will not be able to interact with the target receptor, which is necessary to initiate molecular events to release the viral genome in the cell [76,77]. It would be interesting to test the effects of these monoclonal antibodies on Cx43 hemichannels in follow-up studies [78]. In addition to testing the new set of compounds, it could also be interesting to include other cell systems, such as primary cells, and/or other methodologies to evaluate the effects of the drugs on Cx43 hemichannels [79,80].

## 4. Materials and Methods

### 4.1. Cell Culture Set-Up and Maintenance

DUBCA-hCx43 and HEK-hCx43 cells were thawed and seeded in T75 culture flasks (BD353136, Corning, Glendale, AZ, USA) and maintained with the respective cell culture medium. Dulbecco’s Modified Eagle Medium (DMEM) (21885-025, Gibco, Jenks, OK USA), supplemented with 10% fetal bovine serum (FBS), 1% streptomycin sulfate and 1% sodium benzylpenicillin was used to maintain DUBCA-hCx43 cells. DMEM (11995-065, Gibco, Jenks, OK, USA), supplemented with 10% FBS, 1% streptomycin sulfate, 1% sodium benzylpenicillin and 2 mM L-glutamine was used to maintain HEK-hCx43 cells. Cells were kept in an incubator (37 °C, 5% CO_2_). Cells were seeded either in 6-, 48-, or 96-well plates, and, after 24 h of pre-incubation in cell culture medium (37 °C, 5% CO_2_), they were exposed to azithromycin, chloroquine, dexamethasone, hydroxychloroquine, lopinavir, ribavirin, ritonavir (Sigma-Aldrich, St. Louis, MO, USA), favipiravir (Biosynth Carbosynth, Compton, UK), or remdesivir (Sanbio, Uden, The Netherlands). Stock solutions of the drugs were made in DMSO or water, depending on the solubility, as indicated in Table 1. Then, drugs were dissolved in the corresponding buffer or cell culture medium, as described below. All used drug solutions contained a final concentration of DMSO of 0.6% (Table 1).

### 4.2. Cell Viability Assessment

The viability of the cells was assessed using a MTT assay. In summary, DUBCA-hCx43 cells were seeded in 96-well plates (15,625 cells/cm^2^) and exposed to the drugs for 24 h (37 °C, 5% CO_2_) (Table 1). Drug solutions were made by dissolving the compound in a cell-culture medium. Cells were then incubated (37 °C, 5% CO_2_) with 100 µL MTT solution (0.5 mg/mL MTT (M2128, Sigma-Aldrich, St. Louis, MO, USA) in William’s E medium (A1217601, Thermo Fisher Scientific, Waltham, MA, USA)) for 1.5 h. The MTT solution was aspirated and replaced by 100 µL of DMSO. Plates were shaken on an orbital shaker for 10 min at room temperature to dissolve the formazan crystals in DMSO. Absorbance was measured with a spectrophotometer (VICTOR3^®^ PerkinElmer, Waltham, MA, USA) at 560 ± 10 nm. Viability was expressed relative to untreated control cells, which are cells incubated for 24 h with cell culture medium containing 0.6% DMSO.

### 4.3. Cx43 Hemichannel Activity Assay

The Cx43 hemichannel activity assay was performed as previously described [81]. Briefly, HEK-hCx43 cells were seeded in a 48-well plate (21,053 cells/cm^2^) and pre-incubated for 24 h with cell-culture medium (37 °C, 5% CO_2_). After 24 h, all cells were washed with NC buffer (0.95 mM CaCl_2_ × 2H_2_O, 0.81 mM MgSO_4_ × 7H_2_O, 137 mM NaCl, 5.36 mM KCl, 5.55 mM glucose and 25 mM HEPES in milli-Q water (pH 7.4)). Cells were washed and incubated for 30 min (37 °C, 5% CO_2_) with their corresponding buffer, namely the three control conditions, and different drug solutions. The drugs and Cbx were dissolved in DF buffer (137 mM NaCl, 5.36 mM KCl, 5.55 mM glucose and 25 mM HEPES in mili-Q-water (pH 7.4)). For each drug, a pre-determined range of five concentrations, based on the CC_10_ value, was selected and tested (Table 1). As described earlier, the three control conditions were used to create environments in which the Cx43 hemichannels are stimulated to be in an open (DF) or in a closed (NC and Cbx) state. After an incubation period of 30 min with the corresponding solution, 50 µL of the supernatant of each well was transferred to a white opaque 96-well plate containing 50 µL ATP reaction mixture (ATP Bioluminescent Assay Kit^®^) (FLAA, Sigma-Aldrich, St. Louis, MO, USA) in each well. Luminescence was measured with a plate reader (VICTOR3^®^ PerkinElmer, Waltham, MA, USA). Data were normalized to the DF control.

### 4.4. Immunoblot Analysis

DUBCA-hCx43 cells were seeded in a 6-well plate (15,625 cells/cm^2^) and exposed for 24 h to the drugs (Table 1) dissolved in cell-culture medium (37 °C, 5% CO_2_). The immunoblot analysis procedure was performed as previously described [49,82]. Ice-cold PBS was added to the wells to wash and collect the cells. Following centrifugation, cell pellets were resuspended in lysis buffer and sonicated for 30 s with 50% pulse while keeping the cells on ice. After shaking the samples for 15 min on a rotator at 4 °C, samples were centrifuged at 14,000× *g* for 15 min at 4 °C. A supernatant of each sample was transferred to a new tube and the amount of protein was quantified by means of a bicinchoninic assay according to the manufacturer’s protocol (3225, Thermo Fisher Scientific, Waltham, MA, USA). Following electrophoresis and blotting, nitrocellulose membranes were incubated with 5% non-fatty milk (Régilait, Saint-Martin-Belle-Roche, France) in Tris-buffered saline solution (20 mM Tris and 135 mM sodium chloride) containing 0.1% Tween 20 (Sigma-Aldrich, St. Louis, MO, USA) (TBS-T). Membranes were incubated overnight at 4 °C with primary antibody directed against Cx43 (C6219, Sigma-Aldrich, St. Louis, MO, USA) in a 1:4000 dilution in 5% non-fatty milk in TBS-T, followed by incubation for 1 h at room temperature with polyclonal goat anti-rabbit secondary antibody (P044801-2, Dako, Glostrup, Denmark). Detection of Cx43 was carried out using enhanced chemiluminescence. For semi-quantification purposes, a normalization method based on total protein loading was used to overcome the drawbacks associated with the use of housekeeping proteins [83]. Cx43 signals in DUBCA-hCx43 samples were normalized against total protein loading and expressed as relative alterations compared to untreated DUBCA-hCx43 cells which were considered as control.

### 4.5. RT-qPCR Analysis

DUBCA-hCx43 cells were seeded in a 6-well plate (15,625 cells/cm^2^) and exposed to the drugs for 24 h (37 °C, 5% CO^2^). Total RNA was extracted using a GenEluteTM Mammalian Total RNA purification Miniprep Kit (RTN70-1KT, Sigma-Aldrich, St. Louis, MO, USA) and the On-column DNase I digestion Set (DNASE70, Sigma-Aldrich, St. Louis, MO, USA) according to the manufacturer’s instructions. Isolated RNA was spectrophotometrically measured using a NanoDrop^®^ 2000 Spectrophotometer (Thermo Fisher Scientific, Waltham, MA, USA) to assess purity and quantity. A cut-off ratio between 1.8 and 2.1 for the absorption at 260/280 nm was used for assessing purity. Synthesis and amplification of cDNA, as well as the RT-qPCR analysis, were performed as explained elsewhere [84], with the exception that only 1 µg of total RNA was used to synthesize the cDNA instead of 2 µg. TaqMan probes and primers specific for the target and reference gene are depicted in Table 2. Relative alterations (fold change) in mRNA levels were calculated according to the Pfaffl method [50].

### 4.6. Statistical Analysis

Data were analyzed using a GraphPad Prism 7 (GraphPad^®^ software Inc., San Diego, CA, USA) and were presented as means ± standard deviation. The number of technical replicates (N) and biological replicates (*n*) are specified for each analysis in the figure legends. A parametric one-way analysis of variance followed by a Dunnett’s post hoc test or a Kruskal-Wallis test followed by a Dunn’s multiple comparisons test were used to process the results of the analyses of the experiments depending on the distribution (i.e., Shapiro–Wilk normality test). Outliers were identified via the ROUT’s outlier test and excluded from the data set. Probability (*p*) values ≤ 0.05 were considered statistically significant.

## 5. Conclusions

In summary, the results of this study show that the combination of lopinavir and ritonavir (4:1) as well as remdesivir decreased mRNA levels of Cx43. However, this data could not be supported by immunoblot analysis of Cx43 proteins of cells exposed to these drugs. None of the tested drugs inhibited Cx43 hemichannel activity.

## Figures and Tables

**Table 1:** Panel of drugs and their combinations tested in present study. (DMSO (dimethyl sulfoxide); RT-qPCR (Real-time quantitative reverse transcription polymerase chain reaction analysis); C_max_ (total peak plasma concentration at therapeutic dose) in µM; CC_10_ (concentration inducing 10% cell death)).

Drug	Solvent of Stock Solutions	C_max_ (µM)	CC_10_ (µM)	Concentration Range Tested for CC_10_ Determination (µM)	Concentration Range Tested for Functional Analysis (µM)	Concentration Range Tested for Expression Analysis (µM)	Reference	Supplier
Azithromycin (dihydrate)	DMSO	0.52 [23]	17	1–10–25–50–100–200	1.7–8.5–17–34–170	1.7–8.5–17	A9834	Sigma-Aldrich
Chloroquine (diphosphate)	Water	0.81 [24]	14	1–10–25–50–100–200	1.4–7–14–28–140	1.4–7–14	C6628	Sigma-Aldrich
Dexamethasone	DMSO	0.63 [25]	>200	0.1–0.5–1–10–100–200	0.6–3–6–12–60	0.6–3–6	D4902	Sigma-Aldrich
Favipiravir	Water	53.4 [26]	>400	1–10–25–50–100–200–400	5.3–26.5–53–106–530	5.3–26.5–53	FF29069	Bioynth Carbosynth
Hydroxychloroquine (sulphate)	Water	1.36 [27]	12.5	1–10–25–50–100–200	1.25–6.25–12.5–25–125	1.25–6.25–12.5	HO915	Sigma-Aldrich
Lopinavir	DMSO	19.0 [28]	19	0.25–5–25–50–100–200	1.9–9.5–19–38–190	1.9–9.5–19	SML0491	Sigma-Aldrich
Remdesivir	Water	9.03 [29]	50	0.01–0.1–1–10–50–100	5–25–50–100–500	5–25–50	30354–10	Sanbio
Ribavirin	Water	2.63 [30]	10	1–10–25–50–100–200–400	1–5–10–20–100	1–5–10	R9644	Sigma-Aldrich
Ritonavir	DMSO	1.17 [31]	16	0.25–5–25–50–100–200	1.6–8–16–32–160	1.6–8–16	SML1222	Sigma-Aldrich
Lopinavir:Ritonavir (4:1)	DMSO	19.0 [28]	10:2.5	1–10–25–50–100–200	1:0.25–5:1.25–10:2.5–20:5–100:25	1:0.25–5:1–10:2.5	SML1222 SML0491	Sigma-Aldrich

**Table 2:** Primers and probes used for RT-qPCR analysis of Cx43. (*Gja1*, Cx43; *UBC*, ubiquitinC).

Gene Symbol	Assay ID	Accession Number	Assay Location	Amplicon Size	Accession Number
*UBC*	Hs01871556-s1	M26880.1	2173	135	-
*Gja1*	Hs00748445-s1	NM_000165.5	1031	142	2

## Data Availability

Data are available upon request.
